# The Fast Quantification of Vitamin B12 in Milk Powder by High-Performance Liquid Chromatography-Inductively Coupled Plasma Mass Spectrometry

**DOI:** 10.3390/molecules29081795

**Published:** 2024-04-15

**Authors:** Yue Yang, Biao Zhou, Chenyang Zheng

**Affiliations:** 1 International Healthcare Center, First Affiliated Hospital of Zhejiang University School of Medicine, Hangzhou 310003, China; sarahyuey@163.com; 2 Institute of Nutrition and Food Safety, Zhejiang Provincial Center for Disease Control and Prevention, Hangzhou 310051, China; bzhou@cdc.zj.cn; 3 Institute of Physical-Chemistry and Toxicity, Zhejiang Provincial Center for Disease Control and Prevention, Hangzhou 310051, China

**Keywords:** vitamin B12, cobalt species, milk powder, HPLC-ICP-MS

## Abstract

This study presents a new technique for determining vitamin B12 in milk powder using high-performance liquid chromatography-inductively coupled plasma mass spectrometry (HPLC-ICP-MS). We used ultrasonics with potassium ferrocyanide and zinc acetate solutions to extract the samples. ^59^Co was employed as the analytical target for cyanocobalamin. It was separated using a Phenomenex Luna 5 μm C18 (250 × 4.6 mm) chromatographic column with a mobile phase consisting of 1.6 mmol/L EDTA and 0.4 mmol/L KH_2_PO_4_ in a 60% *v*/*v* methanol solution (pH = 4.0). The sample has an excellent separating degree for free cobalt and cyanocobalamin, and isocratic elution can be finished within 4.0 min. To eliminate the matrix interference due to the presence of milk powder, we applied collision mode (KED). The linear range of cyanocobalamine ranged from 1.0 μg/L to 20 μg/L, with correlation coefficients (r^2^) of 0.9994. The limit of detection (LOD) was 0.63 μg/kg, and the limit of quantitation (LOQ) was 2.11 μg/kg. The mean recoveries were in the range of 87.4–103.6%. The accuracy and precision of the developed method are well suited for the fast quantification of the trace vitamin B12 in milk powder.

## 1. Introduction

Vitamin B12, usually called cobalamin, belongs to a group of cobalt-containing compounds called goblins. The compound can be classified as cyanocobalamin (-CN), hydroxocobalamin (-OH), methylcobalamin (-CH_3_), and adenosylcobalamin (-Ado) based on the various substituents [1]. Humans cannot produce cobalamin on their own and can only obtain it from consuming animal-based foods like meat and milk. Vitamin B12 is crucial for the development of the neurological system, the creation of red blood cells, and the synthesis of fatty acids and specific proteins [2]. Infants, pregnant women, and the elderly are insufficient in B12. This shortage can lead to damage to the blood and nerve systems, hinder cognitive development in infants and children, and heighten fertility risks in pregnant women [3]. The instability of hydroxycobalamin, mecobalamin, and adenosylcobalamin in cobalamin necessitates the use of synthetic cyanocobalamin in milk powder [4,5]. Cyanocobalamin, a compound with a molecular weight of 1355.4, has the cobalt ion bonded to the N-3 of 5, 6-2 methylphenylimidazole above the plane of the cobaltoline ring and to the C5′ of 5′-deoxyadenosine below the plane [6]. The Chinese national standard GB 10765-2021 mandates the addition of vitamin B12 in infant milk powder, with a required content ranging from 0.5 μg/100 g to 7.2 μg/100 g [7]. This requirement aligns with the CODEX STAN 73-1981 standard set by the International Codex Alodex Commission (CAC) for canned baby foods [8].

Currently, the accepted methods for determining vitamin B12 in healthcare supplies are high-performance liquid chromatography (HPLC) [9,10,11,12] and the microbiological method, which is only used for infant food and dairy products [13,14]. Nevertheless, because of the low sensitivity of the HPLC method, it was necessary to enrich and purify it using solid-phase extraction to achieve a higher method detection limit [15,16,17,18]. However, this entire process is intricate and challenging to control. The Association of Official Analytical Chemists (AOAC) employed intricate and time-consuming microbiological methods to determine vitamin B12 in baby food and dairy products [19]. Prior research has utilized the atomic absorption method to indirectly determine vitamin B12 [20]. This method involves enrichment and cleanup using SPE columns to ensure the instrument sensitivity requirements are met. Additionally, cobalt concentration is determined by ICP-MS to indirectly assess the vitamin B12 concentration in samples [21]. In recent years, some research has employed liquid chromatography-mass spectrometry (LC-MS) techniques to determine vitamin B12. Szterk et al. [22] reported a solid-phase extraction (SPE) technique to concentrate vitamin B12 in beef. The concentrated samples were then determined using liquid chromatography-mass spectrometry (HPLC-MS), with a detection limit of 1 μg/kg. Raju et al. [23] reported an HPLC-ICP-MS method for evaluating vitamin B12 in breakfast cereals and multidimensional tables, and the detection limit was 0.9 μg/kg. Liu et al. [24] determined vitamin B12 in functional drinks, multivitamin tablets, and infant milk powder by solid phase extraction column purification and size exclusion chromatography-inductively coupled plasma mass spectrometry (SEC-ICP-MS), and the detection limit was 0.4 μg/L. Yang et al. [1] reported a method that uses microwaves to assist with the extraction of vitamin B12, which is then analyzed using HPLC-ICP-MS.

At present, there are few methods for the determination of vitamin B12 in dairy products without SPE. Moreover, there is no existing research investigating the morphological and chromatographic characteristics of different cobalt species. This study utilized the collision mode (KED) to remove the interference caused by the binding of nitrogen and scandium in the matrix protein of milk powder, as well as the binding of hydroxide and argon in the carrier gas. This was achieved by optimizing the collision reaction parameters [25]. Consequently, the liquid chromatography conditions were optimized to prevent the disruption caused by free cobalt in the presence of cyanocobalamin. An achievable qualitative and quantitative approach using HPLC-ICP-MS was developed to determine vitamin B12 in milk powder. This method may provide technical support in monitoring the nutritional content of milk products.

## 2. Results and Discussion

A tuning solution was employed to adjust the instrument to achieve optimal sensitivity, oxide reduction, double charge correction, resolution, and other indicators of the ICP-MS [24]. The tuned mass spectrum indexes were displayed in Section 3.4. The microwave digestion method was used to process procedural blank and milk powder with a known concentration of vitamin B12. The samples were then measured using ICP-MS, with yttrium used as an internal standard for quantifying ^59^Co. The result was then converted into the concentration of vitamin B12, as shown in Table 1. The concentration of vitamin B12 in the digestion solution of milk powder exceeded the labeled value by approximately 1.85 times. Consequently, the free cobalt in the sample could lead to a false positive result. Hence, the utilization of chromatography is imperative to isolate the free cobalt to achieve a precise determination methodology.

### 2.1. Optimization of Chromatographic Conditions

#### 2.1.1. Selection of Chromatographic Column

**Figure 1:**
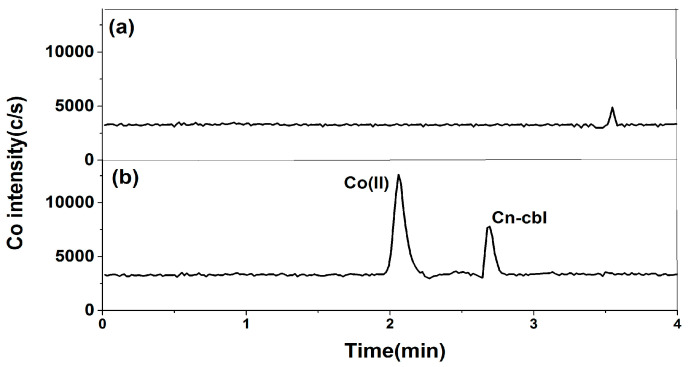
Mixed standard solution of 1 μg/L free cobalt and 20 μg/L cyanocobalamin: (**a**) Diamonsil Plus 5 μm C18 column and (**b**) Phenomenex Luna 5 μm C18 column.

The study used the Diamonsil Plus 5 μm C18 column and the Phenomenex Luna 5 μm C18 column for the reverse phase chromatographic separation of cobalt species. The Diamonsil Plus 5 μm C18 column, a packed column modified with alkyl and polar groups, is appropriate for biomacromolecule analysis. The results showed cobalt species did not retain with the column, along with high column pressure when using EDTA and dihydrogen phosphate as the mobile phase. The analysis was conducted using the Phenomenex Luna 5 μm C18 column, which allowed for the completion of the analysis within 4 min. This column effectively separated free cobalt and cyanocobalamin, as shown in Figure 1.

#### 2.1.2. Selection of Mobile Phase

**Figure 2:**
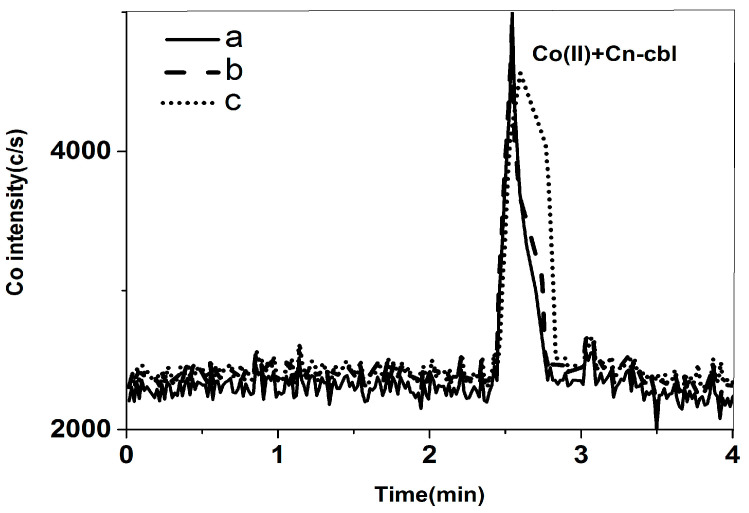
Mixed standard solution of 1 μg/L free cobalt and 20 μg/L cyanobalamine in the mobile phase: (a) 0.4 mmol/L KH_2_PO_4_ in 60% *v*/*v* methanol; (b) 0.8 mmol/L KH_2_PO_4_ in 60% *v*/*v* methanol; (c) 1.2 mmol/L KH_2_PO_4_ in 60% *v*/*v* methanol.

**Figure 3:**
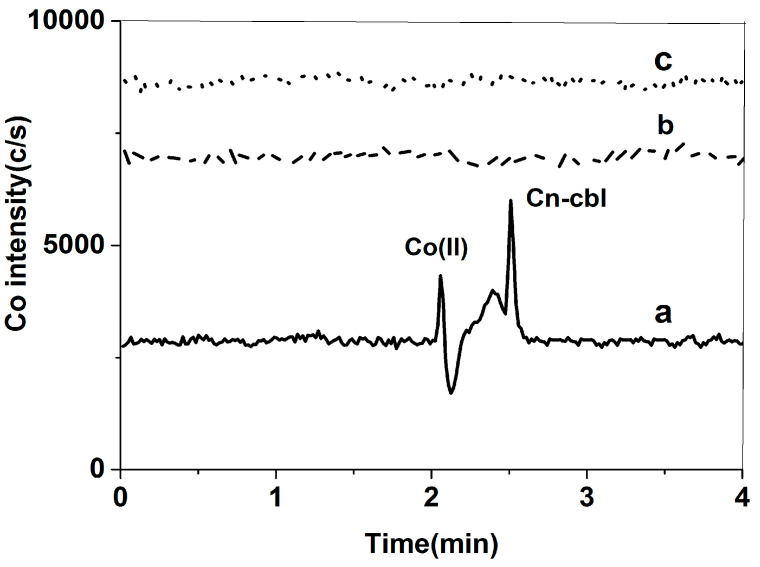
Mixed standard solution of 1 μg/L free cobalt and 20 μg/L cyanobalamine in the mobile phase: (a) 2.0 mmol/L EDTA in 60% *v*/*v* methanol; (b) 5.0 mmol/L EDTA in 60% *v*/*v* methanol; (c) 10.0 mmol/L EDTA in 60% *v*/*v* methanol.

**Figure 4:**
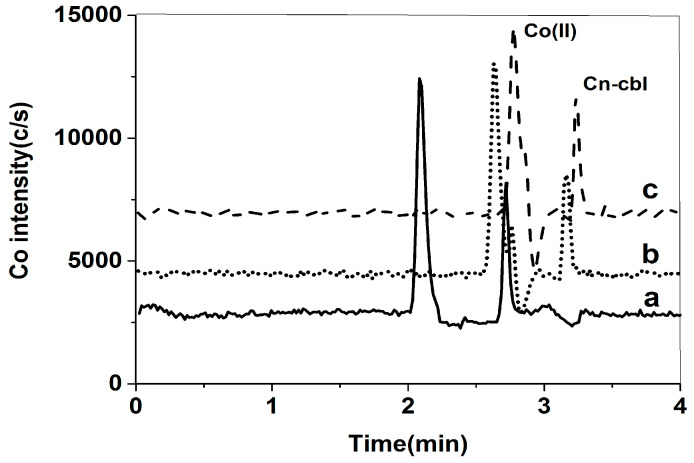
Mixed standard solution of 1 μg/L free cobalt and 20 μg/L cyanobalamine in the mobile phase: (a) 0.4 mmol/L KH_2_PO_4_ and 1.6 mmol/L EDTA in 60% *v*/*v* methanol; (b) 0.4 mmol/L KH_2_PO_4_ and 3.0 mmol/L EDTA in 60% *v*/*v* methanol; (c) 0.4 mmol/L KH_2_PO_4_ and 5.0 mmol/L EDTA in 60% *v*/*v* methanol.

Based on the results mentioned above, it was found that the sample may contain free cobalt, which could impact the measurement of cyanocobalamin. Therefore, it is necessary to eliminate the interference caused by free cobalt. Although removing free cobalt from the extract proved difficult, it is important to note that the concentration of salt in the mobile phase and different solvents can alter the chromatographic properties of both free cobalt and cyanocobalamin. The effect of KH_2_PO_4_ and EDTA on the separation of free cobalt and cyanocobalamin was studied. The concentrations of KH_2_PO_4_ studied were 0.4 mmol/L, 0.8 mmol/L, and 1.2 mmol/L. These concentrations were tested in a solvent consisting of 60% *v*/*v* methanol. The study results revealed that both free cobalt and cyanocobalamin were eluted with the same retention times, and the separation efficacy remained unchanged. The peak pattern observed for the 0.4 mmol/L KH_2_PO_4_ was symmetrical and exhibited a crisp shape, as shown in Figure 2. Furthermore, the concentrations of EDTA that were tested in the solvent of 60% *v*/*v* methanol were 2.0 mmol/L, 5.0 mmol/L, and 10.0 mmol/L. At concentrations of 5.0 mmol/L and 10.0 mmol/L, both free cobalt and cyanocobalamin were retained on the column without any peaks, and the baseline rose as the EDTA concentration increased. Despite the potential enhancement of free cobalt and cyanocobalamin separation by 2.0 mmol/L of EDTA, the sensitivity was low, and the retention time of cyanocobalamin was disrupted by impurity peaks, as shown in Figure 3. Another important function of 0.4 mmol/L KH_2_PO_4_ was to maintain a constant pH of 5.0. A pH of 4.2 was reported to be optimal for the stability of free cobalt and cyanocobalin [23]. In theory, the cobalt species shows effective separation at this pH when the EDTA concentration is appropriately adjusted. Consequently, we decided to use the mixed salt system consisting of KH_2_PO_4_ and EDTA for further selection and optimization experiments. With the addition of 1.6 mmol/L EDTA, 3.0 mmol/L EDTA, and 5.0 mmol/L EDTA into the 60% *v*/*v* methanol mobile phase containing 0.4 mmol/L KH_2_PO_4_, the retention time of free cobalt and cyanobalamin reduced as the concentration of EDTA declined. Additionally, the sensitivity increased. With the addition of 1.6 mmol/L EDTA, the peak shape was symmetrical and sharp (Figure 4). In addition, the study examined the methanol ratios of 40% *v*/*v*, 50% *v*/*v*, and 60% *v*/*v* in the mobile phase. Methanol has the potential to produce symmetrical and sharp peak shapes. As the polarity of the mobile phase decreased due to the increase in methanol, the retention time of free cobalt and cyanobalamin also decreased. The separation degree and sensitivity, however, remained unchanged. It is important to note that if the methanol ratio in the mobile phase exceeds 60% *v*/*v*, the ICP-MS will flame out. To effectively separate cobalt compounds, the experiment used a mobile phase with 0.4 mmol/L KH_2_PO_4_ and 1.6 mmol/L EDTA in a 60% *v*/*v* methanol solution (pH = 4.0).

### 2.2. Optimization of Pretreatment Conditions

**Figure 5:**
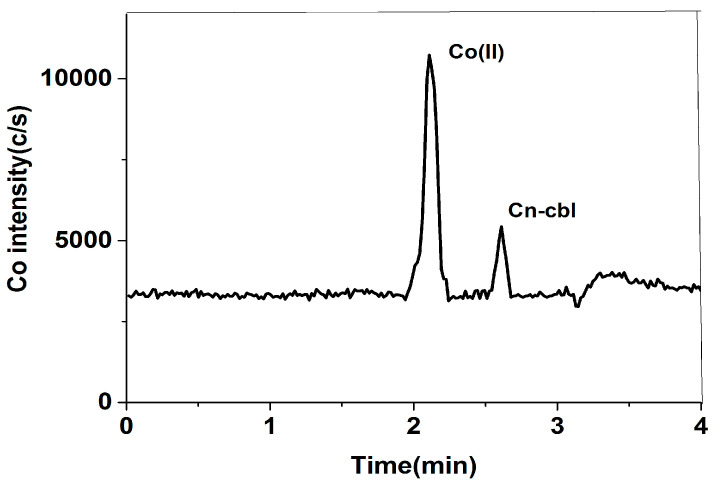
The chromatogram of milk powder sample extract at cell gas B is 1.4 mL/min.

The milk powder sample contained a large amount of protein and organic matter, facilitating the formation of a compound between cyanobalamin and the sample matrix. It contrasts the low adsorption state of cyanobalamin with the matrix in eggs [26]. Using the chromatographic conditions described in Section 2.1, there was a high concentration of unidentified cobalt-containing compounds in the milk powder extract at the same retention time as cyanocobalamin. To clarify the source of interference, the following experiments were designed: (1) Preparing a 1 μg/L cyanocobalamin standard solution using deionized water; (2) Preparing a 1 μg/L cyanocobalamin standard solution using an extraction solution; (3) Spiking cyanocobalamin with a concentration of 1 μg/L to milk powder samples using the extraction solution. Similarly, the three solutions were introduced into ICP-MS, using a cobalt standard solution as a reference, yttrium as an internal standard for quantifying ^59^Co, and finally converted into the concentration of vitamin B12. The experimental results are shown in Table 1. There was no interference in the mass spectrum of deionized water or the extraction solution. However, in the sample of the spiked milk powder, the concentration of vitamin B12 converted from ^59^Co is considerably higher than the known range of vitamin B12 concentration. Cyanobalamin is susceptible to disruption by polyatomic molecules, such as ^14^N^45^Sc, ^43^Ca^16^O^+^, and others. Additionally, the mass spectrum interference from the matrix is highly significant. The collision mode (KED) is a widely used technique in ICP-MS to eliminate the interference caused by mass spectrometry [27,28,29]. The multi-atom interference ions saw a substantial decrease in their kinetic energy upon collision cooling, making it challenging to overcome the potential barrier of the four-stage rod analyzer [30]. The experiment used milk powder extract to examine the optimal collision rate of cell gas B. Finally, when cell gas B was set to 1.4 mL/min, stable and efficient results were achieved (Figure 5). The signal-to-noise ratio (S/N) of the chromatographic peak for cyanobalamin was determined at 2.6 min at the given mass spectrometry setting, with the adjacent noise also being measured. The S/N met the requirements, the BEC was minimized, and the interference caused by the matrix on the chromatographic peak of cyanobalamin was eliminated.

**Figure 6:**
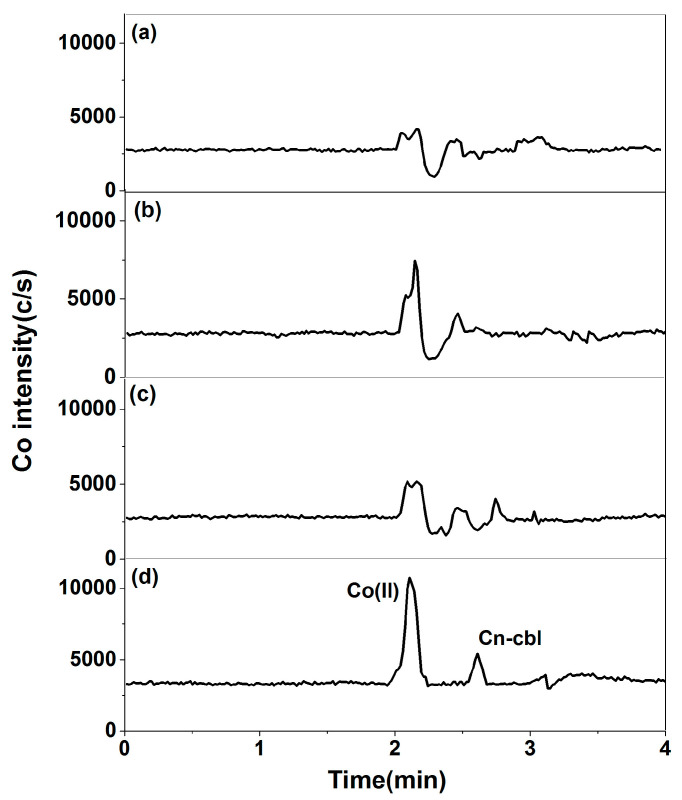
Determination of free cobalt and cyanobalamin in milk powder: (**a**) 60% *v*/*v* methanol; (**b**) 1% *v*/*v* trichloroacetic acid; (**c**) 50 mmol/L sodium acetate buffer solution (pH = 4.0); (**d**) equal volume potassium ferricyanide and zinc acetate solution.

The effects of 60% *v*/*v* methanol, 1% *v*/*v* trichloroacetic acid, 50 mmol/L sodium acetate buffer solution (pH = 4.0), an equal volume of potassium ferrocyanide, and zinc acetate solution as protein precipitators on the determination of cyanobalamin were further investigated (Figure 6). The use of a 60% *v*/*v* methanol solution as a protein precipitator resulted in poor extraction efficiency of cyanobalamin, and a distinct peak corresponding to free cobalt was observed at the retention time. The high acidity of trichloroacetic acid caused the decomposition of cyanobalamin in strong acid, resulting in its loss due to protein precipitation. An impurity peak occurred with a retention time of 2.0 min for free cobalt. When used as a protein precipitator, 50 mmol/L sodium acetate buffer solution exhibited poor peak shape for free cobalt and cyanobalamin, and a split peak was observed during the retention time of free cobalt. Zinc cyanoferrous acid precipitate is formed through the salting-out reaction of zinc acetate and potassium ferrocyanide, following the national food safety standard GB 5009.9-2016 of China [31]. This clarifier is highly effective in eliminating proteins and is perfect for clearing samples with a lower color. The results showed that using potassium ferrocyanide and zinc acetate solutions as precipitators produced a pure extract, leading to an excellent separation between free cobalt and cyanobalamin, exhibiting high sensitivity and minimal interference. Hence, potassium ferrocyanide and zinc acetate solutions can be employed as protein precipitators to isolate cyanobalamin from the impurity peak.

### 2.3. Figures of Merit

The linearity of the calibration curves for free cobalt and cyanocobalamin was evaluated. The area of the chromatographic peak was used for the quantification. The calibration curves for the free cobalt and cyanocobalamin were linear over a concentration range of 0.05 to 1 μg/L and 1 to 20 μg/L with seven calibration points each. The linear regression results are shown in Table 2. The correlation coefficients for free cobalt and cyanocobalamin were found to be 0.9996 and 0.9994, respectively. The limit of detection (LOD) was calculated according to the following equation:

LOD = 3 (N/S) C,(1)

where N represented the baseline noise, S represented the peak height of the measured substance, and C represented the concentration of the measured substance. The limit of quantification (LOQ) was calculated according to the following equation:

LOQ = 10 (N/S) C,(2)

The LOD for free cobalt was 0.045 μg/kg, while for cyanocobalamin it was 0.63 μg/kg, and the LOQ for free cobalt was 0.149 μg/kg, while for cyanocobalamin it was 2.11 μg/kg. The LOD for cyanocobalamin is approximately 15 times better than what is reported in the literature [32]. Six injections of a mixed standard solution including free cobalt and cyanocobalamin at concentrations of 1 μg/L and 20 μg/L were utilized to determine the relative standard deviations (RSD), which were found to be 2.56% and 2.48%, respectively. The results showed that the RSDs (*n* = 6) of cobalt species were less than 5%, indicating that the calibration curves show high precision.

### 2.4. Accuracy of Method

We analyzed certified reference materials for quality control purposes and procedure blanks to assess potential sources of contamination. National Standard Substances (GBW 10277) obtained from the National Institute of Metrology (NIM, Beijing, China) were analyzed for quality control of cyanocobalamin. The results are shown in Table 3. It can be found that there is a good agreement between the found and certified values (t-test at a 95% confidence level). Since there are no certified reference materials for free cobalt in milk powder, the impact of the entire experimental process, including extraction and detection, may be verified through the recovery of the spiked samples [29]. The results were listed in Table 4. The recoveries of free cobalt and cyanocobalamin were 94.5% to 102.0% and 87.4% to 103.6%, respectively. The RSDr for the analysis (six injections for three spike levels) were within acceptable limits (less than 5%), showing that this analysis method was repeatable and reliable in milk powder samples.

### 2.5. Analysis of Real Samples

A technique was employed to determine the vitamin B12 content in six kinds of infant milk powder. The vitamin B12 concentration in milk powder met the range of 0.5–7.2 μg/100 g, as specified in the CODEX STAN 73-1981 Standard for canned baby food. The total cobalt species concentration determined by HPLC-ICP-MS was consistent with the ^59^Co obtained by ICP-MS following microwave digestion. The cobalt species concentration accounted for 89.9–97.5% of the total cobalt. The results were listed in Table 5. Compared to previous methods, this method could determine the cobalt species with simple pretreatment, reduced time requirements, and excellent reliability.

## 3. Materials and Methods

### 3.1. Sample Source

The milk powder samples were collected from brand milk powder available at the shopping mall. The collected samples have been confirmed to contain cyanocobalamin as a supplement to vitamin B12 and should be stored in a dark place [33].

### 3.2. Instruments and Reagents

The following instruments and reagents were used in this experiment: NexION 300D ICP-MS (PerkinElmer, Waltham, MA, USA) equipped with a concentric nebulizer and collision reaction cell; Series 200 HPLC (Perkin Elmer, Waltham, MA, USA); de-iron water was acquired using a Millipore water purifier (Millipore Ltd., Bedford, MA, USA); Phenomenex Luna 5 μm C18 (250 × 4.6 mm) column; ETH031 Microwave Digestion Instrument (Milestone Co., Roseland, NJ, USA); KQ-500DE Numerical Control Ultrasonic Cleaner (Kunshan Instrument Co., Shanghai, China); high-speed centrifuge (Eppendorf Co., Hamburg, Germany); cobalt standard solution (1000 mg/L) (SPEX Co., Metuchen, NJ, USA); vitamin B12 (Cyanobalamin, Sigma-Aldrich Co., Burlington, MA, USA); infant milk powder standard substances GBW 10277 (NIM, Beijing, China); nitric acid and methanol (chromatographic purity, Merck, Darmstadt, Germany); ethylenediamine tetraacetic acid disodium (EDTA), potassium dihydrogen phosphate, disodium hydrogen phosphate dodecahydrate, potassium ferrocyanide, anhydrous sodium acetate, trichloroacetic acid, zinc acetate, and acetic acid (analytical purity, Chinese Medicine Co., Beijing, China).

### 3.3. Chromatographic Conditions of HPLC

ICP-MS did not tolerate the organic mobile phase in the absence of an oxygenation device. The excessive presence of organic solvents in the injection solution can lead to the flameout of ICP-MS and result in the accumulation of carbon deposits in the cone system, thereby impacting the accuracy of the experimental results [34]. Hence, the quartz center tube of the Perkin Elmer PFA Base, coupled with an oxygenation device, was used for this experiment. The mobile phase consisted of EDTA and dihydrogen phosphate salt. However, a high concentration of EDTA might lead to salt deposition at several critical points in the liquid system, such as the one-way valve, sampling cone, and intercepting cone of the ICP-MS instrument [1]. Therefore, selecting a low concentration of EDTA is preferable to protect the instrument. Thus, the Phenomenex Luna 5 μm C18 (250 × 4.6 mm) column was employed. The mobile phase consisted of a solution containing 1.6 mmol/L of EDTA and 0.4 mmol/L of potassium dihydrogen phosphate in a 60% *v*/*v* methanol solution (pH = 4.0). Isostatic elution was used, with an injection volume of 20 μL and a column flow rate of 1.0 mL/min.

### 3.4. Mass Spectrometry Conditions of ICP-MS

For optimal operation of the KED, parameters including Rpq and cell gas B are critical. By utilizing the value of the bandpass parameter Rpq, which is 0.45, the proper RF voltage for quadrupole rods can be determined. The collision gas flow rate of cell gas B was 1.4 mL/min. The mass number *m*/*z* = 58.9 (Co) was monitored, while the atomizer had a flow rate of 0.82 mL/min. The RF power was 1350 W. The flow rate of the plasma gas was 18.0 mL/min, while the flow rate of oxygen was 0.065 mL/min. The auxiliary gas was flowing at a rate of 1.2 mL/min.

### 3.5. Method Validation

The method was validated in terms of linearity, LOD, LOQ, recovery, and repeatability. To evaluate the linearity, the mixed standard solutions of free cobalt (0, 0.05, 0.1, 0.2, 0.5, 0.8, and 1.0 μg/L) and cyanocobalamin (0, 1.0, 2.0, 5.0, 8.0, 10.0, and 20.0 μg/L) were prepared for seven-point calibration curves. The correlation coefficients were evaluated to a fit of at least 0.999. Previous studies have investigated the LOD and LOQ using different statistical methods, such as the standard deviation of the response and the slope, to determine these values. The equation LOD = 3.3 σ/S and LOQ = 10 σ/S is employed in the objective, where σ represents the standard deviation of the response and S indicates the slope of the calibration curve [35]. Another approach includes determining the concentrations that produce a signal (peak area) three or ten times greater than the sample matrix blank in the chromatogram [36]. In order to determine the sensitivity of the LOD and LOQ, it is essential to utilize a sample matrix devoid of cyanocobalamin. Due to the cyanocobalamin contained in the milk powder, it is essential to dilute the sample extract. However, the matrix effect has alterations when the sample matrix is diluted, resulting in unrepresentative results for the LOD and LOQ. Consequently, the lowest calibration concentration that provides a chromatographic signal with a signal-to-noise (S/N) ratio of 3 or 10, respectively, is determined to be the LOD and LOQ. The accuracy of the method was assessed by analyzing certified reference substances and determining the percentage of cobalt species in the spiked milk powder. The milk powder was spiked with free cobalt at concentrations of 0.5, 1.0, and 4.0 μg/kg and with cyanocobalamin at concentrations of 10, 25, and 50 μg/kg. Six replicates were analyzed for each concentration level. Repeatability was determined by monitoring the RSDr (intra-day precision) for the spiked samples at each spiking level on the same day; each sample was measured six times.

### 3.6. Sample Experiments

Precipitants for protein were prepared by dissolving 106 g of potassium ferrocyanide in deionized water and diluting it to a volume of 1000 mL. Similarly, 220 g of zinc acetate was dissolved in deionized water, along with 30 mL of acetic acid, and diluted to a volume of 1000 mL [31]. A 2.0 g (accurate to 0.0001 g) sample was weighed. Subsequently, 1.0 mL of potassium ferrocyanide solution and 1.0 mL of zinc acetate solution were introduced, and 8 mL of deionized water was added. The solution was ultrasonicized at a temperature of 30 ℃ for 40 min and centrifuged at a speed of 4000 r/min for 3 min. The supernatant was filtered through a 0.20 μm hydrophilic membrane before its injection into the HPLC-ICP-MS system. Simultaneously, each set of samples was processed alongside a procedural blank, determined using the HPLC-ICP-MS method. The blank effectively eliminated false positives caused by contamination in the extraction process, instrument, or chemicals. The samples were also digested completely using a pressurized microwave digestion procedure using nitric acid. The milk powder samples (0.3 g) were weighed into closed Teflon PFA vessels. Moreover, 5 mL of nitric acid was added, and the vessels were heated in a microwave digestion instrument to decompose the sample. The microwave digestion program was as follows: (1) ramp time of 40 min, power of 1300 W, and temperature of 185 °C; (2) hold time of 30 min, power of 1300 W, and temperature of 185 °C. After cooling, the digests were transferred to a 15 mL centrifuge tube, diluted with deionized water to reach the volume, and subsequently introduced into the ICP-MS for total cobalt concentration analysis. Three procedural blanks were prepared for each digestion run using an acid matrix identical to that used for sample preparation.

## 4. Conclusions

This study developed an analytical method using HPLC-ICP-MS to determine vitamin B12 in milk powder samples. ^59^Co was employed as the analytical target for cyanocobalamin. It was separated using a Phenomenex Luna 5 μm C18 (250 × 4.6 mm) chromatographic column, with a mobile phase consisting of 1.6 mmol/L EDTA and 0.4 mmol/L KH_2_PO_4_ in a 60% *v*/*v* methanol solution (pH = 4.0). Two cobalt species were determined within 4 min. An equal volume of potassium ferrocyanide and zinc acetate solutions were used as protein precipitators. The LOD for vitamin B12 was 0.63 μg/kg, whereas the LOQ was 2.11 μg/kg. This method provides a fast, simple, and highly sensitive separation technique for cobalt species in milk powder. Additionally, it fulfills the standard detection criteria for vitamin B12 in milk powder samples. The developed technique can also be applied to quantify trace amounts of cobalamin in different food samples.

## Figures and Tables

**Table 1:** Mass spectrometry interference of cobalt in four experiments measured by ICP-MS.

Four Experiences Designed	Concentration Found (μg/L)
Cyanobalamin standard with water	0.03
Cyanobalamin standard with extract solution	0.02
Sample extracted	40.48
Sample digested	5.55
Sample blank digested	0.00

**Table 2:** Methods linear range, with the LOD and LOQ.

Cobalt Species	Linear Equation	Linear Range (μg/L)	Correlation Coefficient (r^2^)	LOD (μg/kg)	LOQ (μg/kg)	RSD (%)
Free cobalt	y = 50,130x + 0.6239	0.05–1.0	0.9996	0.045	0.149	2.56%
Cyanocobalamin	y = 1863.6x + 194.79	1–20	0.9994	0.63	2.11	2.48%

**Table 3:** Analysis of standard reference substances ^a^.

	GBW 10277	
Species	Found (μg/L)	Certified (μg/L)
cyanocobalamin	30.3 ± 0.7	29.8 ± 3.6

^a^ mean values and 95% confidence intervals (n = 6).

**Table 4:** Concentrations of cobalt species and recoveries in milk powder as measured by HPLC–ICP-MS ^a^ (n = 6).

Sample	Background Value (μg/kg)		Added (μg/kg)	Concentration Found (μg/kg)	Mean Recovery (%)	RSDr (%)
Milk powder	Co(II)	2.1	0.5	2.5 ± 0.1	95.3	4.42
			1.0	3.1 ± 0.1	99.6	3.10
			4.0	6.2 ± 0.2	101.3	2.76
	Cyanocobalamin	27.3	10	33.8 ± 2.1	90.6	4.66
			25	53.4 ± 1.5	102.2	4.01
			50	79.7 ± 2.8	103.1	2.98

^a^ Values are the means of six measurements ± standard deviation.

**Table 5:** Concentrations of cobalt species and total cobalt in six milk powders ^a^.

	Sample 1	Sample 2	Sample 3	Sample 4	Sample 5	Sample 6
Cyanocobalamin	27.3	14.7	28.5	10.6	33.7	16.9
Sum ^b^	29.4	16.1	30.3	11.3	37.1	19.5
Digested ^c^	30.9	17.9	32.3	12.1	40.9	20.0
The ratio of cobalt species and total cobalt (%)	95.1	89.9	93.8	93.4	90.7	97.5

^a^ All values are in μg/kg units unless otherwise noted; ^b^ Sum of concentration of individual cobalt species obtained by HPLC–ICP-MS; ^c^ Total concentration of cobalt in the pressurized microwave digests.

## Data Availability

Data are contained within the article.
